# Pharmacopœia of the United States, Published by Authority of the National Medical Convention Held at Washington

**Published:** 1831

**Authors:** 


Art. IX. The Pharmacopœia of the United States of America. *Published by authority of the National Medical Convention, held at Washington, A. D.*, 1830. *Philadelphia: John Grigg*, 1831. p. 268, Oct.

That every civilized country in which medical science is cultivated, should have a standard authority, in the shape of a Pharmacopœia, seems now to be universally conceded, and although the faculty in the United States have been slow to imitate the example of other countries in relation to this matter, we have ample reason to believe, that this department of medical literature will hereafter be duly appreciated.

It is matter of grief, however, that any circumstances should have occurred, calculated to produce the least diversity of opinion or practice, in relation to a book, which, from its very title, ought to possess an uniform influence. We are sorry to witness any thing like collision in an affair, which should, of all others, be harmonious in its operations. A national work, for national benefit, should have the honor of the nation as one of the strongest incentives to its formation, and all local jealousies should vanish and be forgotten. Never can the medical profession in this country, nor in any other, rise in importance and in true dignity, until all its members, individually agree to sacrifice something for the good of the whole. Local prejudices, and narrow minded, sectarian views must be laid aside, and our watchword must ever be *union*, and *honorable emulation*. Our reference is now, specially, to a work recently issued from the press of New-York, and purporting to be the long-looked for National Pharmacopœia. We have only to say, in respect of that production, that it bears internal, as well as external evidence, whatever its authors may think of it, that it is *not* such a work, as the wants of the country demanded. We are perfectly willing to submit it, however, as well as the book now under brief review, to the consideration and final decision of the public.

It is not our intention to examine, in detail, the work before us; our principal aim being to call the attention of the medical profession to it, and through that medium, to prevail upon our apothecaries, to give it a fair and impartial examination.

There are parts to which, as individuals, we feel disposed to take exception; but we are not inclined to insist on our peculiar views, as a ground of objection, to what, on the whole, we regard as the best production of the sort, which has ever met our eye. Of some articles introduced into the Pharmacopœia, we should say, that the book and the profession would be better without them; and of one or two others, that more felicitous modes of pharmaceutical management might have been detailed. In the present state of things, we are of opinion, that the fewer public objections, especially, if they be trivial in their nature, the better for the final result. Let us accept of the offering as it is presented to us, and by a repeated examination, ascertain all its weak parts, and a suitable period will arrive, in which to avail ourselves of the best application of the result of our researches. The revision of the national work once in ten years, is a circumstance which offers every inducement that can be desired, to analyze thoroughly and carefully the whole system, and to suggest whether changes may be regarded as important or necessary. We are not ignorant of the difficulties attendant on the formation of a National Pharmacopœia, and not only here, but in foreign countries; and we know, that not a work of this sort has ever yet been sent from the press, that did not excite, whether justly or unjustly we care not, the sneers of some, the sarcasm of others, and the approbation of a goodly number.

Our desire, frankly expressed, is, that the book may find entrance into every medical library, and into every apothecary's shop in our country. We are moved to the expression of this desire by two reasons; the first is, that the attention of all who, are interested, professionally, in the perfection of such a work, may be excited and deeply concentrated in it, and the other is, that a most indefatigable and worthy publisher may receive, in some measure, the patronage he so richly merits.

We cannot dismiss the book before us, without hazarding in a future edition, one of our peculiarities, at least. The days in which we live, are somewhat revolutionary in their character, and the signs of the times indicate, that a period is approaching, in which it will be fashionable to practice medicine, without incurring the sin of promoting intemperance. We firmly believe, that spirituous potations are not essential to the cure of disease, and therefore that tinctures need no longer to have a conspicuous place in our pharmaceutical systems. If one physician can practice, unaided by the powers of alcohol, it is not assuming too much to aver, that the entire profession could, if they chose, dispense with that article, as an internal medicine. And we think, that a greater degree of responsibility rests on our profession in respect to the temperance reformation, than on any other class in the community; and that the duty we owe the public, can be most efficiently discharged, by affixing the seal of entire abstinence on our national Pharmacopœia.

